# Effect of Real-time Feedback on the Quality of Cardiopulmonary Resuscitation for Medical Students in Colombia

**DOI:** 10.5811/westjem.50647

**Published:** 2026-07-31

**Authors:** Jose Luis Piñeros-Alvarez, Nelson Esteban Portuguez-Jaramillo, Astrid Lorena Urbano-Cano

**Affiliations:** * Universidad Santiago de Cali, Grupo de investigación en Genética, Fisiología y Metabolismo - GEFIME, Universidad Santiago de Cali, Cali, Colombia; † Universidad del Valle School of Medicine, Research Group in Prehospital Care, Emergencies and Disasters, Cali, Valle del Cauca, Colombia; ‡ Universidad Santiago de Cali, Grupo de Investigación en Salud Integral (GISI), Departamento Facultad de Salud, Universidad Santiago de Cali, Cali, Colombia

## Abstract

**Introduction:**

High-quality cardiopulmonary resuscitation (CPR) is essential to improve survival in cases of cardiac arrest. However, skill acquisition based solely on instructor observation has proven to be limited. In this context, real-time feedback devices emerge as promising tools to optimize CPR training.

**Objective:**

In this study our goal was to evaluate the effect of using a real-time feedback device on the quality of CPR performed by medical students at a public university in Colombia.

**Methods:**

This was an experimental study with fourth-year medical students, randomly assigned to two groups. Both groups received the same initial theoretical and practical training in basic CPR. Subsequently, the intervention group practiced with a real-time feedback device, while the control group trained without technological feedback. The primary outcome measure was the target of chest compressions, simultaneously meeting both a rate of 100–120 compressions/minute and a depth of 5–6 cm (maximum value: 100%), as well as ventilations delivered within the 500–600 mL volume range. Secondary outcomes included isolated data on compression depth and rate during two simulated scenarios: hands-only CPR for two minutes and five cycles of 30 chest compressions and two ventilations.

**Results:**

Of 106 eligible participants, 98 (92.5%) completed the test (46 intervention / 52 control). Regarding the primary outcome, the intervention group showed significantly higher compliance with the compression target compared to the control group (median values: intervention group 31% vs control group 0%, *P* < .001). In the hands-only CPR test, the intervention group achieved a higher compression target (72% [40.7–81] vs 3.4% [0–40]; *P* < .05) and better depth control (5.5 cm [5–5.6] vs 6 cm [5.6–6.1]); *P* < .05], with no differences in rate (*P* = .71). In the test with cycles of 30 chest compressions and two ventilations, the intervention group performed better, with a higher compression target (73% [5–83] vs 0.5% [0–19]), P< .05) and better depth control (5.5 cm [5–5.8] vs 6 cm [5.9–6.1], P < .05). No significant differences were observed in ventilation. Both groups had a median target completion of 0% with tidal volumes (intervention group, 265 mL; control group, 291 mL), accounting for failure at well below the recommended 500–600 mL.

**Conclusion:**

Real-time feedback significantly improved chest compression quality in medical students, although ventilation difficulties persisted. Future research is needed to assess long-term skill retention and clinical application.

## INTRODUCTION

Cardiac arrest remains a significant public health challenge worldwide. Despite advances in resuscitation care, survival rates remain low for both out-of-hospital and in-hospital cardiac arrests.^1^–^3^ Outcome variability after cardiac arrest is significantly influenced by modifiable resuscitation factors, particularly cardiopulmonary resuscitation (CPR) quality metrics such as compression depth, rate, chest recoil, and minimization of interruptions.^4^,^5^ International guidelines emphasize the importance of effective resuscitation training to improve survival rates in these scenarios.^6^,^7^ However, the progressive decline of CPR skills following training continues to be a concern,^8^–^10^ with inconsistencies in CPR quality even among trained personnel.^8^,^9^

Traditionally, medical students undergo CPR training using simulators with instructor-provided feedback, who guide and correct performance according to quality parameters. Although this method has demonstrated satisfactory learning outcomes in some studies, it is generally considered less effective than technology-assisted teaching methods, which allow real-time feedback on quality parameters.^11^–^12^ Furthermore, it has been reported that instructor judgment alone is insufficient to determine student competence in chest compressions.^13^ Although the use of feedback devices has been shown to improve outcomes, and most studies support their inclusion in healthcare training, many institutions still do not integrate them into their teaching models.^14^–^17^

With the release of updated international guidelines, specific quality parameters have been established to optimize resuscitation effectiveness.^18^ Real-time feedback devices now enable more accurate measurement of these parameters, generating objective data that allow immediate adjustments in technique.^19^ Recently, there has been growing interest in incorporating these devices into CPR education, as they help to overcome the limitations of subjective visual assessment by instructors and healthcare staff.^20^ Previous research has shown that feedback devices can improve short-term learning outcomes.^20^–^22^

Training in CPR is essential in medical education, as it is crucial to assess the acquisition of high-quality skills and identify areas for improvement to strengthen knowledge.^23^,^24^ Therefore, we sought to assess the impact of real-time feedback devices on the quality of CPR performed by fourth-year medical students.

## MATERIALS AND METHODS

### Study Design

A randomized, cross-sectional, single-blind study was conducted at Universidad del Valle, Cali, Colombia, from May 5–30, 2025. The study was registered and approved by the institutional ethics committee (code 013–024) and the School of Medicine. Participation was confirmed through signed informed consent, and all data were handled to ensure student anonymity.

**Population Health Research Capsule**

What do we already know about this issue?

*High-quality cardiopulmonary resuscitation is crucial for improving survival rates in cardiac arrest*.

What was the research question?

*Does the use of a real-time feedback device during training improve the rate and depth of chest compressions and ventilation volume?*

What was the major finding of the study?

*Training with real-time feedback significantly improved the quality of chest compressions but did not improve ventilation quality*.

How does this improve population health?

*This research supports the integration of real-time feedback technology in medical education*.

### Participants

The medical program offers a mandatory annual course titled “Basic Cardiopulmonary Resuscitation Techniques” for fourth-year students prior to initiating their clinical rotations. Recruitment was carried out through email announcements and calls made by the academic office immediately before the course began. Inclusion criteria were being of legal age and officially enrolled in the course. Exclusion criteria included the following: chronic cardiac or respiratory diseases; pregnancy; motor disabilities that would significantly limit performance of the tests; and language barriers.

### Randomization

Each participant was assigned a numerical code. A researcher external to the study generated a list of these codes in a digital spreadsheet, randomized the order using the “random” function, and allocated participants into two groups with a 1:1 ratio to ensure balance. The first group was designated CPR with feedback and the second CPR without feedback. Participants were notified of their group assignment but were blinded to the characteristics of the testing protocol.

### Sample Size Calculation

We calculated the sample size based on previous data^25^ using the following parameters: a confidence level of 95% (α = 0.05), a statistical power of 80% (1 - β = 0.20), and an estimated minimum difference of 20% in the proportion of high-quality CPR between groups (effect size). An additional 10% was added to account for potential losses (dropouts), resulting in a final sample size of 94 participants.

### Feedback Device

During the study, a single PRESTAN CPR 2000 adult simulator (PRESTAN Products, LLC, Solon, OH) was used. It was purchased at a cost of U.S. dollars 400. This device provides visual cues through light indicators on the thoracic side and auditory signals during compressions. It is equipped with software that records and displays real-time performance via wireless connection to mobile devices or monitors through the PRESTAN CPR feedback app. Data can be exported for quantitative analysis, including compression rate and depth, number of ventilations, ventilation volume, and pause duration, among others. The simulator establishes an upper compression depth limit of 6.1 cm, with any measurement beyond this value representing excessive compression depth.

### Protocol

The training program, based on the latest International Liaison Committee on Resuscitation guidelines,^23^,^24^,^26^ consisted of four four-hour weekly sessions. The first session covered theoretical aspects of adult and pediatric CPR, as well as automated external defibrillator use. The subsequent sessions were organized into practical skills stations in the laboratory. The principal investigator met with instructors beforehand to standardize the teaching pathway according to the algorithms in the guidelines,^26^ ensuring homogeneous training conditions for all participants.

The intervention group (CPR with feedback) trained with the feedback device, allowing them to view CPR variables in real time and adjust performance immediately during practice. The control group (CPR without feedback) received the same training in a separate space, without the use of feedback devices. To minimize bias, both groups trained using PRESTAN simulators.

At the end of the course, participants from both groups were summoned for the study protocol. In the intervention group, students were paired (“A” and “B”). The protocol had two phases: in phase one, participant “A” performed continuous compressions for two minutes without ventilations, followed by participant “B.” A mandatory five-minute rest was provided to minimize fatigue-related confounding. In phase two, each pair completed five CPR cycles (30 compressions followed by 2 ventilations). Within each pair, participant “A” performed chest compressions, while participant “B” delivered ventilations with a bag-valve-mask (BVM). After completing the five cycles, participants switched roles and repeated the test.

Before starting, participants were allowed to familiarize themselves with the simulator and had two minutes of practice for technical adjustment. An investigator explained the simulator’s features and feedback tools and answered questions. During phase one, a tablet was placed on the floor in front of the participant; during phase two, it was placed diagonally to allow each participant to track their own performance. Two investigators were present at all times: one supervised and provided instructions, while the other transcribed data exported from the device (Figure 1). The control group followed the same protocol, but the simulator’s feedback lights were covered with opaque tape, and no feedback devices were used.

For data collection, a test was considered complete if both phases of the protocol were performed. It was considered incomplete if a participant missed more than one theoretical-practical session or failed to complete one of the two protocol activities.

### Primary and Secondary Outcome Measures

For the hands-only CPR test, we defined the primary outcome as the target of compressions, calculated as the absolute percentage of optimal chest compressions that simultaneously met both a rate of 100–120 compressions/minute and a depth of 5–6 cm.^27^,^28^ For the test with cycles of 30 chest compressions and 2 ventilations, the outcomes included the total target of compressions across all five cycles, as well as the cycle target of compressions for each individual cycle. Additionally, the total target of ventilations and the cycle target of ventilations were determined. The ventilation target was defined as the percentage of effective ventilations based on an administered volume of 500–600 mL.^29^ Secondary outcomes for all tests included the following:: the proportion of compressions within the recommended depth (5–6 cm); chest compressions performed at the recommended rate (100–120 compressions/minute); “hands-off time” defined as the interruption period without chest compressions during rescue ventilations; and the total CPR time in the modality using cycles of 30 chest compressions and 2 ventilations.

### Statistical Analysis

We analyzed all data using IBM SPSS software v25 for Windows (International Business Machines Corporation, Armonk, NY). Frequency distributions were calculated for categorical variables, and results were expressed as percentages. To assess the distribution of continuous variables, we applied the Kolmogorov-Smirnov test with Lilliefors correction. Variables with normal distribution were reported as means with standard deviations, whereas non-normally distributed data were described using medians and interquartile ranges, obtained through the Mann-Whitney U test. We assessed statistical significance using the Fisher exact test and the chi-square test.

## RESULTS

A total of 98 students agreed to participate in the study, of whom 57 (58.2%) were women. The predominant age range was 18–25 years (89.8%). Additionally, most participants had never undergone any type of CPR training or external certification prior to completing this university course (92.8%). Of the total included participants, 52 were assigned to the control group and 46 to the intervention group. The evaluation of sociodemographic variables showed no significant differences between groups (Table 1).

For the hands-only CPR test, statistically significant differences were found in the compression target: intervention group, 72% (40.7–81) vs control group, 3.4% (0–40) (*P* < .05), indicating greater accuracy in the simultaneous achievement of recommended compression depth and rate in the intervention group. When analyzing the median compression depth separately, the intervention group achieved 5.5 cm (5–5.6) compared to the control group’s 6 cm (5.6–6.1 (P < .05), showing significant differences, with the intervention group remaining within the recommended range of 5–6 cm. No significant differences were found in compression rate, as both groups remained within the recommended range: intervention group 111.5 (106–115) vs control group 111 (108–114); P = .71 (Table 2).

In the test with cycles of 30 chest compressions and 2 ventilations, the total compression target was significantly higher in the intervention group compared to the control group; intervention group 73% (5–83) vs control group 0.5% (0–19); *P* < .05. No significant differences were found between groups in the total ventilation target: intervention group 0% (0–22.5) vs control group 0% (0–20); *P* = .99. Overall, the administered tidal volumes were below the recommended range in both groups: intervention group 265 mL (206–390) vs control group: 291 ml (219–373); P =.990, suggesting substantial difficulty in delivering effective ventilations.

The median compression depth demonstrated better control in the intervention group: 5.5 cm (5–5.8) vs control group 6 cm (5.9–6.1); *P* < .05. Compression rate remained within the recommended range in both groups: intervention group 109 (106–114) vs control group 111 (107–114); *P* = .544. The mean hands-off time was lower in the control group, with significant differences, indicating fewer interruptions in chest compressions: intervention group 6.0 (1.4) vs control group 4.8 (0.79); *P* < .05. Likewise, the mean total time to complete five cycles was longer in the intervention group 111.6 seconds (10.85) vs control group 107.4 (6.45); *P* < .05 (Table 2).

Cycle-by-cycle analysis showed that the median compression target remained consistently higher for the intervention group compared with the control group. No significant differences were observed in ventilation. Both groups had a median target completion of 0% and tidal volumes (intervention group 265 mL; control group 291 mL), well below the recommended 500–600 mL. Compression depth remained within the recommended 5–6 cm range throughout all cycles in the intervention group compared with the control group. Finally, a modest variability in compression rate was observed in the intervention group, while the control group showed more consistency, although without negative impact, as values always remained within the recommended range (Table 2, Figure 2).

No data loss, transcription errors, or software-related issues were reported. Full follow-up of all participants was ensured, and there were no exclusions due to incomplete protocol performance.

## DISCUSSION

We evaluated whether the use of real-time feedback devices would improve CPR performance among fourth-year medical students. Quality guidelines for CPR are as follows: in adults, chest compressions should reach a depth between 5–6 cm; be delivered at a rate of 100–120 compressions per minute; ensure complete chest recoil after each compression; minimize interruptions during CPR; and avoid hyperventilation.^19^ Proper implementation of these measures has been associated with increased survival to hospital discharge in patients with cardiac arrest.^7^,^30^

Our findings showed that both chest compression depth and compliance with the compression target were significantly higher in the group trained with real-time feedback devices. In a study conducted in 30 hospitals in the United States with healthcare workers, it was concluded that chest compressions are often suboptimal and that the use of feedback devices improves compression quality in simulators.^31^ These results are relevant because current guidelines emphasize the importance of achieving chest compressions that simultaneously meet both adequate depth and rate, proposing this combined measure as an objective criterion to evaluate compression quality.^27^

Many educational institutions rely on the instructor’s subjective assessment to evaluate CPR quality. However, visual assessment has been shown to be highly inaccurate, even among experienced healthcare professionals.^21^ This leads to a false perception of CPR quality and causes clinicians to perform suboptimal CPR during simulated cardiac arrest scenarios.^22^ In this context, the lack of objective assessment during CPR training may negatively influence skill development.

The low performance of the control group in the primary outcome (simultaneous compression target) could be explained by the strictness of the composite metric. While participants often achieved adequate depth or rate individually, maintaining both parameters simultaneously proved difficult for them without real-time feedback guidance. A systematic review published in 2021 reported that the use of real-time feedback devices, regardless of the specific type, can guide both laypersons and professionals in achieving high-quality CPR targets.^17^ This supports the use of automated devices as a strategy to improve performance in simulated settings. Consistent with the results presented in our study, the intervention group demonstrated higher compliance values for quality variables compared to the control group.

Ventilation is considered a key component of CPR because it facilitates gas exchange. Current guidelines recommend providing 2 ventilations for every 30 compressions when an advanced airway is not available, with a tidal volume of 500–600 mL, or enough to produce visible chest rise.^19^ Recent publications have shown that despite efforts to achieve guideline-consistent ventilations, rescuers often fail to deliver adequate volumes with BVM, usually providing volumes below the recommended threshold.^32^ In our study, both groups delivered low tidal volumes, resulting in zero compliance with ventilation quality targets.

These findings differ from other reports in simulated settings where ventilation quality was assessed in 20 pairs of paramedics. That study found that real-time ventilation feedback improved compliance with ventilation rate targets (41–71%) and volume targets (31%79%).^33^ Typically, in the absence of objective markers, healthcare professionals rely on observing chest rise as an indicator of adequate ventilation volume.^34^ An observational study with 106 simulated, adult cardiac arrest resuscitations showed that ventilation practices rarely met guideline targets, even when performed by well-trained professionals.^35^ Simulation-based studies suggest that the use of feedback devices may significantly enhance the ability of professionals to achieve recommended ventilation parameters during simulated cardiac arrest resuscitations.^36^

However, it is noteworthy that in our study, the intervention group, despite having real-time feedback devices, demonstrated ventilation performance far below recommendations, similar to that of the control group. Possible explanations include difficulties in BVM technique, mask leaks, or ineffective airway alignment with the simulator. Previous studies have shown that BVM ventilation performed by two rescuers is more effective than when performed by a single individual.^37^ The most common deficiency in the latter is ineffective mask sealing,^38^ a situation observed in most of our participants. One explanation for the widespread failure to meet ventilation parameters suggests that the difficulty lies in the psychomotor skill required to maintain an effective mask seal and airway patency, rather than in the lack of feedback alone.

We must emphasize that feedback devices that measure tidal volume also present limitations, such as their inability to reflect respiratory system physiology, including airway resistance or lung compliance. Therefore, developing methods to monitor and ensure high-quality ventilation during CPR education is crucial. Furthermore, it should be recognized that performance with a feedback device may reflect a “calibration” to the specific mechanical compliance of the manikin, which does not fully replicate the variable chest stiffness encountered in real patients.

Interruptions in compressions may reduce coronary and cerebral perfusion pressures and have been associated with worse outcomes when interruption time is prolonged.^39^ During CPR with cycles of 30 chest compressions and 2 ventilations, compressions are briefly interrupted to allow ventilations. The optimal duration of these interruptions remains uncertain.^40^ International guidelines recommend that interruptions should not exceed 10 seconds, and recent studies indicate better clinical outcomes with shorter interruptions.^41^ In our study, the mean “hands-off” time was 4.8 seconds for the control group and 6 seconds for the intervention group, with a statistically significant difference. These findings are consistent with other studies where experienced rescuers required between 5–7 seconds to perform this task.^32^,^41^ However, as noted earlier, the tidal volumes delivered were suboptimal.

The study population consisted of fourth-year medical students, 92.85% of whom had never participated in activities related to life support and had limited experience in psychomotor skills that require practice to achieve proficiency. Real-time audiovisual feedback devices allow immediate monitoring of key parameters, facilitating adherence to CPR quality goals in simulation scenarios.^19^ Therefore, incorporating such tools into CPR training may be useful to optimize skill acquisition during educational sessions. A variety of simulators featuring real-time feedback are available on the market. Regarding practicality, the device used represents a cost-effective alternative for low-resource settings compared to high-fidelity simulation centers.

## LIMITATIONS

Future studies should evaluate longer CPR cycles, more closely resembling real-life scenarios, including rescuer switching and assessment of additional variables such as chest compression fraction and chest recoil. These represent some of the limitations of the present study, which also include constraints inherent to the feedback device used. Various devices are available in the market, each with different mechanisms for data acquisition. For example, the equipment used in this study does not allow quantitative analysis of chest recoil within its software. Similarly, compression depth has an upper limit of 6.1 cm, even if the participant exceeds this value. It would be important to compare different feedback devices and assess the similarity or discrepancy among data outputs. Additionally, due to the design of our study, no longitudinal follow-up was performed to evaluate skill retention over time. Finally, it is important to recognize that the simulator is strictly a training tool and not a clinical device. Therefore, the operationalization of improved classroom skills into real-world clinical practice and their direct impact on patient outcomes remain to be evaluated.

## CONCLUSION

The results demonstrate that the application of real-time feedback devices for training medical students in CPR significantly improved chest compression quality during a simulated scenario. However, despite the use of such devices, participants showed difficulty in achieving ventilation targets. Therefore, educational strategies should be directed toward optimizing ventilation techniques to match the improvements observed in compression quality. Additionally, future research is needed to assess long-term skill retention, clinical application in real patients, and comparison between different clinical feedback devices.

### Ethics Approval and Consent to Participate

We confirm that all methods were carried out in accordance with the ethical standards set forth in the Declaration of Helsinki and its subsequent amendments, or comparable ethical standards. The study was registered and approved by the institutional ethics committee (code 013–024) and the School of Medicine. Participation was confirmed through signed informed consent, and all data was handled to ensure student anonymity.

## Figures and Tables

**Figure 1:**
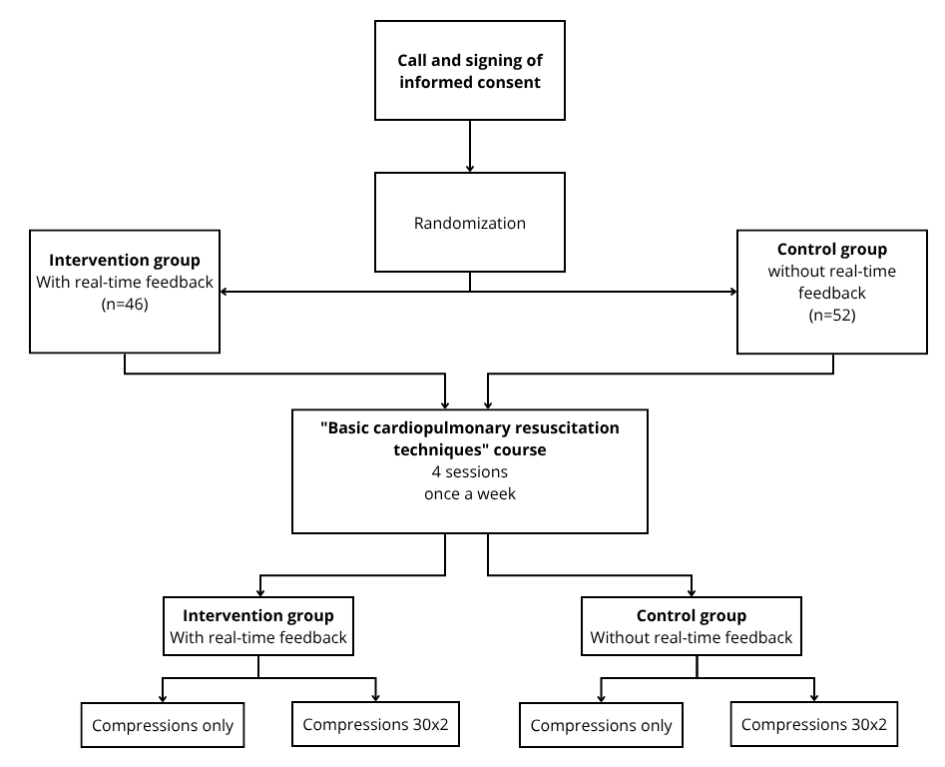
Flowchart illustrating participant selection and protocol development process in a study of real-time feedback on quality of cardiopulmonary resuscitation among medical students in Colombia. *Note:* 30x2 compressions refers to cycles of 30 compressions followed by 2 ventilations.

**Figure 2:**
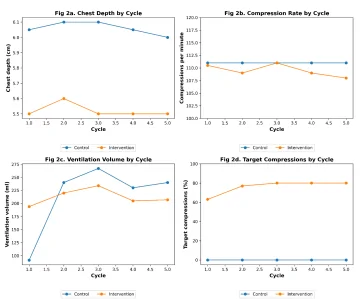
Monitoring of compliance with cardiopulmonary resuscitation (CPR) quality criteria (depth, rate, ventilation, and target compressions) during each cycle, stratified by groups in a study of real-time feedback on CPR quality among medical students in Colombia. *Note:* target compressions, percentage of compressions simultaneously meeting correct depth and rate. With regard to chest depth, the feedback device records a maximum depth of 6.1 cm; therefore, values at this level indicate a depth of ≥ 6.1 cm.

**Table 1:** Sociodemographic variables of participants categorized by sex, age, and previous attendance in cardiopulmonary resuscitation (CPR) courses in a study of real-time feedback on CPR quality among medical students in Colombia.

Variable n (%)	Control n = 52	Intervention n = 46	P value
Sex			
Female	31 (59.6)	26 (56.5)	.84
Male	21 (40.4)	20 (43.5)	
Age			
18–25 years	48 (92.3)	40 (87)	.51
26–33 years	4 (7.7)	6 (13)	
External CPR Courses			
6–24 months ago	1 (1.9)	1 (2.2)	
> 24 months ago	3 (5.8)	2 (4.3)	1
No external courses	48 (92.3)	43 (93.5)	

*CPR*, cardiopulmonary resuscitation

**Table 2:** Comparison of compliance with cardiopulmonary resuscitation (CPR) quality criteria in compression-only and 30:2 modalities (including cycle-specific performance) between intervention and control groups in a study of real-time feedback on CPR quality among medical students in Colombia.

Modality		Quality Criteria Median (IQR)		Control, N=52	Intervention, N=46	P value
Compression only		Target compressions (%)		3.4 (0 – 40)	72 (40.7 – 81)	.00
		Chest depth (cm)		6 (5.6 – 6.1)	5.5 (5 – 5.6)	.00
		Compressions per minute		111 (108 – 114)	111.5 (106 – 115)	.71
Cycles of 30 chest compressions and 2 ventilations		Target compressions (%)		0.5 ( – 19)	73 (5 – 8)	.00
		Target ventilations		0 ( – 20)	0 ( – 22.5)	.99
		Chest depth (cm)		6 (5.9 – 6,1)	5.5 (5 – 5.8)	.00
		Compressions per minute		111 (107 – 114)	109 (106 – 114)	.54
		Ventilation volume (mL)		291 (219 – 374)	265 (206 – 390)	.99
		Hands-off time (sec)		4.8 (SD 0.8)	6.0 (DE ± 1.4)	.00
		Total CPR time (sec)		107.4 (SD 6.4)	111.63 (DE ± 10.8)	.00

Cycles		Cycles of 30 chest compressions and 2 ventilations (modality by cycle)				

		Target compressions (%)	Target ventilations (%)	Chest depth (cm)	Compressions per minute	Ventilation Volume (mL)

Control, N=52	1	0 (0 – 12)	0 (0 – 0)	6.0 (6 – 6.1)	111 (107 – 114)	91.5 (0 – 330)
	2	0 (0 – 9)	0 (0 – 0)	6.1 (6 – 6.1)	111 (107 – 114)	240 (0 – 366)
	3	0 (0 – 7)	0 (0 – 0)	6.1 (5.9 – 6.1)	111 (108 – 114)	267 (47 – 374)
	4	0 (0 – 20)	0 (0 – 0)	6.0 (5.9 – 6.1)	111 (106 – 114)	230 (0 – 370)
	5	0 (0 – 26)	0 (0 – 0)	6 (5.9 – 6.1)	111 (107 – 114)	240 (0 – 334)
Intervention, N=46	1	63 (19.7 – 83)	0 (0 – 0)	5.5 (5.3 – 5.9)	110.5 (104 – 116)	194 (0 – 366)
	2	77 (41 – 87)	0 (0 – 0)	5.6 (5.3 – 5.8)	109 (106 – 115)	220 (0 – 371)
	3	80 (52 – 90)	0 (0 – 0)	5.5 (5.3 – 5.7)	111 (105 – 114)	234 (0 –363)
	4	80 (53 – 87)	0 (0 – 0)	5.5 (5.3 – 5.7)	109 (105 – 115)	205 (0 – 374)
	5	80 (67 – 90)	0 (0 – 0)	5.5 (5.3 – 5.7)	108 (104 – 114,2)	207 (0 – 391)

Data are presented as median (interquartile range) for continuous variables due to non-normal distribution, and as mean (standard deviation) for time variables. Target compressions: percentage of compressions simultaneously meeting correct depth and rate. Target ventilations: percentage of ventilations with tidal volume between 500–600 mL. *P* value was calculated using the Mann-Whitney U test for continuous variables. The feedback device records a maximum depth of 6.1 cm.; therefore, values reported as 6.1 cm represent depths ≥ 6.1 cm.

*SD*, standard deviation.
